# Generation and immunogenicity analysis of recombinant classical swine fever virus glycoprotein E2 and E^rns^ expressed in baculovirus expression system

**DOI:** 10.1186/s12985-021-01507-1

**Published:** 2021-02-24

**Authors:** Qiang Wei, Yilin Bai, Yapeng Song, Yunchao Liu, Wei Yu, Yaning Sun, Li Wang, Ruiguang Deng, Guangxu Xing, Gaiping Zhang

**Affiliations:** 1 Henan Provincial Key Laboratory of Animal Immunology, Henan Academy of Agricultural Sciences, Zhengzhou, 450002 China; 2 College of Veterinary Medicine, Northwest A&F University, Yangling, 712100 China; 3 College of Animal Science and Veterinary Medicine, Henan Agricultural University, Zhengzhou, 450002 Henan China; 4 Henan Baiao Biological Project Co., Ltd., Zhengzhou, 450002 China; 5 Jiangsu Co-innovation Center for Prevention and Control of Important Animal Infectious Diseases and Zoonoses, College of Veterinary Medicine, Yangzhou University, Yangzhou, 225009 Jiangsu Province China

**Keywords:** Classical swine fever virus, Immunogenicity, E2, E^rns^

## Abstract

Classical swine fever (CSF) caused by the classical swine fever virus (CSFV) is a highly contagious swine disease resulting in large economical losses worldwide. The viral envelope glycoprotein E2 and E^rns^ are major targets for eliciting antibodies against CSFV in infected animals. In this report, the glycoprotein E2 and E^rns^ were expressed using the baculovirus system and their protective immunity in rabbits were tested. Twenty CSFV seronegative rabbits were randomly divided into five groups. Each rabbit was intramuscularly immunized with CSFV-E2, CSFV-E^rns^, or their combination (CSFV-E2 + E^rns^). Besides, a commercial CSFV vaccine (C-strain) and PBS were used as positive or negative controls, respectively. Four weeks after the second immunization, all the rabbits were challenged with 100 RID_50_ of CSFV C-strain. High levels of CSFV E2-specific antibody, neutralizing antibody and cellular immune responses to CSFV were elicited in the rabbits inoculated with C-strain, CSFV-E2, and CSFV-E2 + E^rns^. And the rabbits inoculated with the three vaccines received complete protection against CSFV C-strain. However, no neutralizing antibody was detected in the E^rns^ vaccinated rabbits and the rabbits exhibited fever typical of CSFV, suggesting the E^rns^ alone is not able to induce a protective immune response. Taken together, while the E^rns^ could not confer protection against CSFV, E2 and E2 + E^rns^ could not only elicit humoral and cell-mediated immune responses but also confer complete protection against CSFV C-strain in rabbits.

## Introduction

Classical swine fever (CSF) is a highly contagious and economically important viral disease of swine, that is notifiable to the World Organization for Animal Health [1]. The causative agent, classical swine fever virus (CSFV) is a member of the genus *Pestivirus* within the family *Flaviviridae*. The CSFV positive-stranded RNA genome consists of a single long open reading frame encoding a polyprotein of approximately 3900 amino acids. The polyprotein is cleavage processed by cellular and viral proteases to the mature forms of final viral proteins. The viral proteins include four structural proteins (the nucleocapsid protein C, E1, E2, and E^rns^) and eight nonstructural proteins (p7, NS2, NS3, NS4A, NS4B, NS5A, NS5B, and N^pro^) [2].

There are three envelope glycoproteins in the CSFV virion, E^rns^, E1, and E2. E^rns^ is loosely associated with mature virions [3] and possesses ribonuclease activity [4]. It is heavily glycosylated with carbohydrate moieties at seven *N*-linked glycosylation sites and glycosylation contributes to nearly half of the molecular mass of the protein [5–7]. E2 is a major determinant of CSFV virulence and is involved in virus attachment and entry into target cells [8, 9]. It is shown that glycosylation plays a major role in the immunogenicity of CSFV envelope proteins [7]. In addition, previous studies demonstrate that both E2 and E^rns^ are capable of inducing the production of neutralizing antibodies in the host [10–12].

The modified live virus (MLV) vaccines based on C-strain are effective and provide complete protection against the CSFV. However, the conventional vaccines could not offer differentiability of infected from vaccinated animals. Thus, subunit vaccines are designed to meet the DIVA (differentiation of infected from vaccinated animals) requirement for vaccination. The two CSFV envelope glycoproteins E^rns^ and E2 have been targeted for subunit vaccine development. Most of the studies have been focused on the E2 and the protein is well recognized as the protective antigen [13–17]. In contrast, the effectiveness of E^rns^ as a vaccine target has been controversial in different studies. It is shown that single administration of purified E^rns^ glycoprotein could induce an effective protection against CSFV infection in a previous study [7]. However, another study showed that recombinant E^rns^ expressed in yeast *P. pastoris* could not induce neutralizing antibody and protection against CSFV infection [18]. Thus, further investigation is needed to evaluate the immunogenicity of the protein and its ability to induce protection against CSFV.

In the present study, two recombinant baculoviruses were constructed to separately express E2 and E^rns^. The glycosylation of the two protein was analyzed and the protective immune responses of the recombinant protein E2, E^rns^ or E2 + E^rns^ to CSFV were investigated in a rabbit model.

## Materials and methods

### Cell line and virus strain

Porcine kidney cells (PK-15) were grown and maintained in DMEM (Gibco) supplemented with 10% fetal bovine serum (Hyclone) and 1% Penicillin–streptomycin solution (Gibco). *Spodoptera frugiperda* 21 insect cells (sf21) were grown in Sf-900II medium at 27 °C. The commercial vaccine (C-strain) was purchased from GuangDong YongShun Biological Co., Ltd (China) for immunization and challenge in the animal experiments. CSFV Shimen strain was passaged four times in PK-15 cells cultured in DMEM supplemented with 10% FBS in order to be used for the serum neutralization test and the lymphocyte proliferation assay.

### Plasmid construction

For secretory expression of the protein, the gp67 signal peptide was inserted into the vector pFastBac1 with homologous recombination method using the following primers: F: 5′-CCACCATCGGGCGCGGATCTATGCTACTAGTAAATCAGTCACACCAA-

GGCTTCAATAAGGAACACACAAGCAAGATGGTAAGCGC-3′ and R: 5′-GCAA-

GATGGTAAGCGCTATTGTTTTATATGTGCTTTTGGCGGCGGCGGCGCATTCTGCCTTTGCGGGATCCCGGTCCGAAG-3′. The resulting plasmid was designated as pFastBac1-gp67. For secretory expression of E2, the C-terminal transmembrane region was deleted [19–21]. Consequently, the coding sequence for the amino acid residues 1–331 of E2 was cloned into the pFastBac1-gp67, with a C-terminal 6 × His tag fused to the fragment. The E^rns^ gene (the amino acid residues 1-190) containing the mainly antigenic region [22–24] was also cloned into the pFastBac1-gp67, with a C-terminal 6 × His tag fused to the fragment. This truncation was meant to avoid aggregation during secretion and purification due to the amphipathic helix at the C terminus that is inserted slightly tilted into the membrane [25–27].

### Protein expression and purification

The recombinant plasmids were transformed into *Escherichia coli* DH10Bac. Upon screening of colonies, positive colonies were subcultured in LB broth. And the recombinant bacmid of E2 or E^rns^ was isolated using Qiagen plasmid mini kit. Then the recombinant baculovirus was generated using the Bac-to-Bac system (Invitrogen). Sf21 cells were infected for large-scale protein production. The cell cultures were collected and clarified by centrifugation at 9000 × *g* for 20 min. E2 and E^rns^ protein were purified using the nickel-affinity chromatography followed by size-exclusion chromatography (HiLoad 16/60, Superdex 200; GE Healthcare). The proteins were verified by SDS-PAGE and subsequently by western blot using anti-His antibody. The protein concentration was quantified with a Micro BCA™ protein assay kit 120 (Pierce Biotechnology, USA).

### Preparation of vaccines

The CSFV subunit vaccines, CSFV-E2 and CSFV-E^rns^ were produced by mixing the antigen with ISA 201 adjuvant (Seppic, France) at a ratio of 50:50 (w/w) according to the manufacturer’s instructions (100 μg of the E2 or E^rns^ protein per 2 mL). The subunit vaccine CSFV-E2 + E^rns^ containing 100 μg of E2 protein and 100 μg of E^rns^ protein per 2 mL was manufactured with the same method.

### Vaccination and challenge in rabbits

Twenty 14-week-old female New Zealand white rabbits (Hualan Biological Engineering INC, Xinxiang, China) were randomly divided into 5 groups (n = 4). Groups A–C were immunized with 2 mL of CSFV-E2, CSFV-E^rns^, and CSFV-E2 + E^rns^, respectively. Group D was inoculated with 100 RID_50_ (50% rabbit infectious dose) of the commercial C-strain vaccine as positive control. Group E was inoculated with 2 mL of PBS as negative control. Booster immunization was given at the same dose at 4 weeks post primary immunization. All the rabbits were intravenously challenged with 100 RID_50_ of CSFV (C-strain) at 4 weeks after booster immunization. After challenge, rectal temperatures were recorded every 12 h to detect the typical fever of rabbits. A typical fever is characterized by a ≥ 1 °C increase in rectal temperature for a minimum of 18 h.

### Measurement of anti-E2 and anti-E^rns^ antibodies using ELISA assays

The presence of E2-specific antibody was determined by blocking ELISA with IDEXX CSFV antibody test kit (IDEXX GmbH, Switzerland) according to the manufacturer’s instructions. All samples were tested in triplicate and the optical density were measured at 450 nm (OD_450_) with an ELISA plate reader (POLARstar Omega, Germany). The mean OD_450_ of the negative controls was more than 0.5 and the mean blocking rate of the positive controls (${\text{NC}}\bar{\text{x}}$) was greater than 50%, thus the assay was considered valid. The results were reported as the antibody blocking rate. The blocking rate was calculated with the equation, blocking rate =  $100 \times \frac{{{\text{NC}}\bar{\text{x}} - {\text{SampleOD}}_{{450}} }}{{\text{NC}}\bar{\text{x}}}$. A blocking rate ≤ 30% was interpreted as negative, a blocking rate between 30 and 40% was interpreted as suspected, and a rate ≥ 40% was interpreted as positive.

Anti-E^rns^ antibodies were determined by ELISA. Briefly, the 96-well flat-bottomed microtiter plates were coated overnight at 4 °C with 10 μg/mL of purified E^rns^ protein (100 μL/well) in coating buffer (0.1 M carbonate buffer, pH 9.6). Subsequently, the plates were washed with PBST (PBS containing 0.05% Tween-20) for three times. Next, the serum samples (50 μL /well, in duplicate) were added to the plates and incubated for 1 h at room temperature. Then, horseradish peroxidase (HRP)-conjugated goat anti-rabbit IgG (Jackson ImmunoResearch, West Grove, PA, USA) was used as secondary antibody. The plates were developed using TMB stabilized chromogen, and the reactions were stopped with 2 N sulfuric acid. Relative antibody concentrations were determined using the ELISA plate reader (POLARstar Omega, Germany) at 450 nm.

### Serum neutralization test

The serum neutralization test was performed to detect CSFV antibodies as previously described [16]. Briefly, the serum samples were inactivated for 30 min at 56 °C. They were then serially diluted two-fold with PBS. The diluted serum samples (in duplicate) were incubated with 100 TCID_50_ of CSFV Shimen strain for 1 h at 37 °C in 5% CO_2_. Residual virus infectivity was determined by adding 1.0 × 10^4^ PK-15 cells to each well and incubated at 37 °C in 5% CO_2_ for 3 days. Subsequently, the cells were subjected to immunoperoxidase monolayer assay (IPMA). Firstly, the cells were fixed with methanol containing 3% H_2_O_2_ at room temperature for 10 min. After washing three times with PBST, cells were incubated with blocking buffer containing 5% skim milk at 4 °C overnight. Then, cells were incubated with hyperimmune porcine CSFV antiserum (prepared in our lab) and followed by HRP-conjugated goat anti-swine IgG (Jackson ImmunoResearch, West Grove, PA, USA). Finally, cells were stained with AEC solution (ZSGB-BIO, Beijing, China) at room temperature and examined by light microscopy. Samples of CSFV positive reference serum (Institute of Veterinary Drug Supervision of China, Beijing, China) and CSFV-negative serum (prepared in our lab) were used as control samples. The titers of CSFV specific neutralization antibodies were expressed as the reciprocal of the highest dilution that caused 50% neutralization.

### Lymphocyte proliferation assay

Peripheral blood mononuclear cells (PBMCs) were isolated from the immunized rabbits at 49 days post immunization using the rabbit lymphocyte isolation kit (Solarbio, China) and resuspended in RPMI 1640 medium containing 10% FBS. The cells (1 × 10^6^ cells/mL) were seeded into 96-well plates with 100 μL per well and then stimulated with CSFV Shimen strain or unstimulated. All treatments were performed in triplicate. After 72 h of incubation, WST-8 (10 μL/well, Beyotime Biotechnology, China) was added and incubated for a further 1 h at 37 °C. The absorbance of each well was measured using the ELISA plate reader (POLARstar Omega, Germany) at 450 nm. The results were expressed as stimulation index (SI) = mean of the OD_450_ values of the CSFV-stimulated wells/mean of the OD_450_ values of CSFV-unstimulated wells.

### Analysis of IFN-γ and IL-4 mRNA expression

PBMCs were cultured in 12-well plates for 48 h with or without the stimulation of CSFV Shimen strain. Total RNA was extracted and reverse transcribed in a 20 µL reaction mixture. The cDNA product was further amplified using a SYBR Green Master (Roche) and specific primers (IFN-γ F: CTGGTCCAGCGTAAAGCAGT and R: TCAGTACTTGGATGCTCGCC; IL-4 F: CAGGGGCGACATCATCCTAC and R: CTCGGTTGTGTTCTTGGGG; GAPDH F: AGAGCACCAGAGGAGGACG and R: TGGGATGGAAACTGTGAAGAG) as previously described [16]. Real-time RT-PCR amplification was performed using the following cycling parameters: 10 min at 94 °C, and 40 cycles of 15 s at 94 °C and 60 °C for 30 s. Each experiment was performed in triplicate. In summary, gene expression was determined by relative quantity, which compares the threshold cycle (Ct) of the sample of interest (CSFV-stimulated PBMCs) to the Ct generated by a reference sample (non-stimulated PBMCs). Take IFN-γ as an example, the IFN-γ gene expression was normalized to GAPDH expression by subtraction of Ct to obtain ΔCt values. The ΔΔCt was calculated as the difference between ΔCt values for CSFV-stimulated and non-stimulated PBMCs. The relative difference in IFN-γ expression between stimulated and unstimulated cells was determined using the equation $2^{{ - \Delta \Delta {\text{Ct}}}}$ according to the User Bulletin number 2, ABI prism 7500 Sequence Detection System (Applied Biosystems) instructions.

### Detection of CSFV RNA through RT-NestPCR assay

The rabbits were euthanized at 4 days after challenge and the spleens were collected under sterile conditions. The total RNA was extracted and reverse transcribed. For RT-NestPCR [28], cDNAs were prepared for first round PCR with outer primer pairs (Outer-F: 5′-CAACTGGCTAGTTAATGCA-3′ and Outer-R: 5′-AATGAGTGTAGTG-

TGGTAAC-3′) in a 25 μL cocktail containing 2 × rTaq mix (Takara) 12.5 μL, 10 μM Outer-F or Outer-R primer 1 μL, double distilled water 8 μL and cDNA 2.5 μL. The first round PCR steps are as following: initial denaturation at 95 °C for 1 min, 25 cycles at 94 °C for 30 s, 54 °C for 30 s and 72 °C for 30 s, followed by a final extension at 72 °C for 10 min. The first round PCR products were then used as templates for second round PCR with inner primer pairs (Inner-F: 5′-ATGATGATGACCCTGATA-3′ and Inner-R 5′-GTGTGGTAACTTGAGGTAG -3′) in a 25 μL cocktail containing 2 × rTaq mix 12.5 μL, 10 mM Inner-F or Inner-R primer 1.5 μL, double distilled water 9 μL and first round PCR product 0.5 μL. The second round PCR steps are as following: initial denaturation at 95 °C for 3 min, 35 cycles at 94 °C for 30 s, 56 °C for 30 s and 72 °C for 20 s, followed by a final extension at 72 °C for 10 min. Finally, the amplified products were analyzed by electrophoresis.

### Statistical analysis

Comparisons and graphs were analyzed using GraphPad Prism version 7.0. Differences were considered statistically significant at *p* < 0.05.

## Results

### Expression and purification of recombinant proteins E2 and E^rns^

**Fig. 1:**
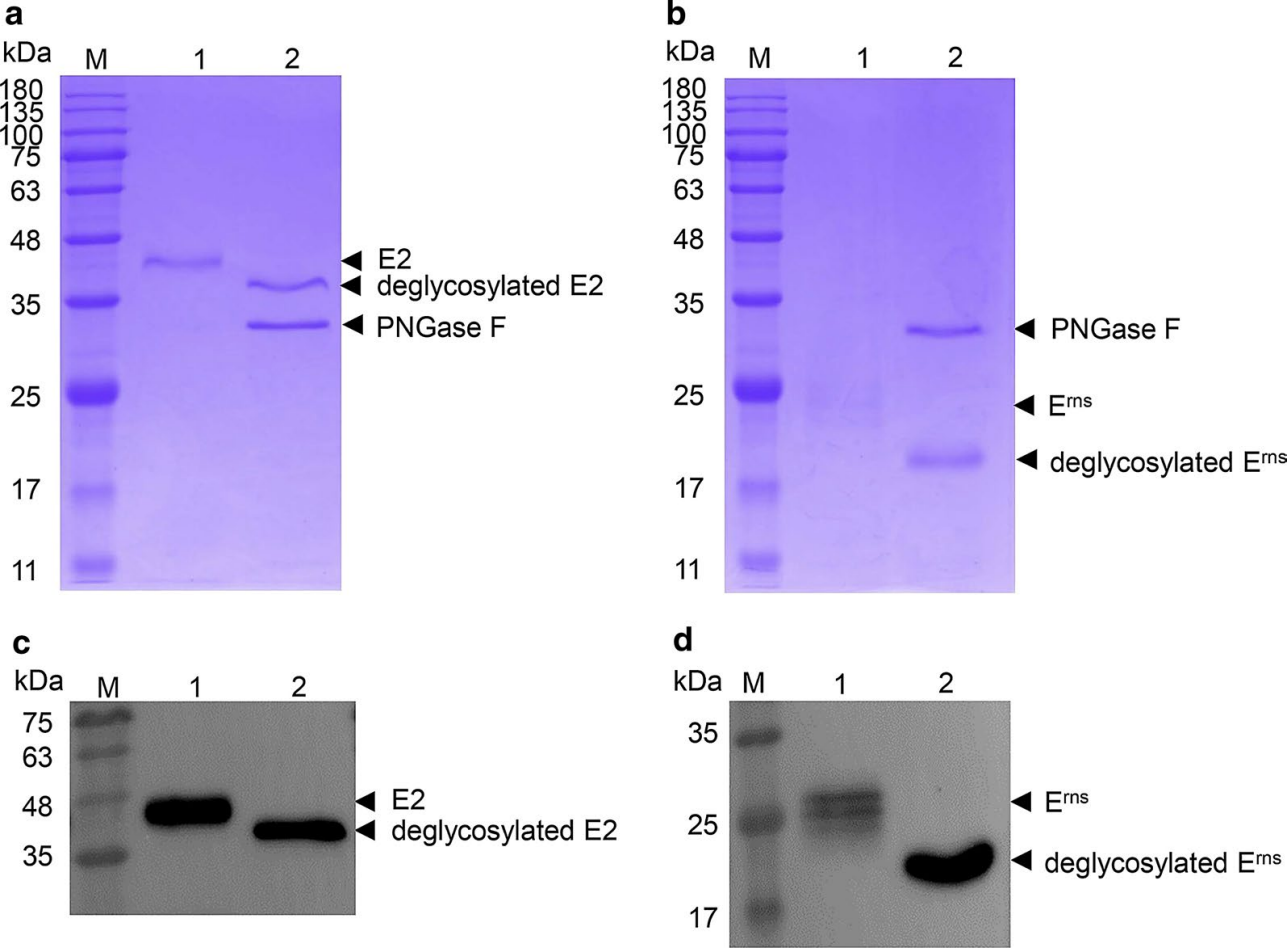
Expression and characterization of recombinant CSFV E2 and E^rns^ protein. **a** SDS-PAGE analysis of E2 protein. Lane 1, purified E2 protein; Lane 2, E2 protein treated with PNGase F. **b** SDS-PAGE analysis of E^rns^ protein. Lane 1, purified E^rns^ protein; Lane 2, E^rns^ protein treated with PNGase F. **c** Western blot of purified E2 protein. Anti-His antibody was used for the western blot. Lane 1, purified E2 protein; Lane 2, E2 protein treated with PNGase F. **d** Western blot of purified E^rns^ protein. Anti-His antibody was used for the western blot. Lane 1, purified E^rns^ protein; Lane 2, E^rns^ protein treated with PNGase F

CSFV E2 or E^rns^ gene from the CSFV shimen strain was cloned into the recombinant baculovirus and the recombinant proteins were expressed using sf21 insect cells. The culture medium was then collected by centrifugation. And the supernatant was subjected to protein purification with Ni-NTA agarose beads. As shown in Fig. 1, recombinant E2 and E^rns^ protein were purified from the culture medium. The recombinant E2 protein appeared to be 45 kDa, and the recombinant E^rns^ was about 25 kDa. The bands were diffuse and even multiple bands were observed in the analysis of E^rns^ protein. Moreover, the proteins ran at higher molecular weight than predicted mass calculated from the amino acid composition, indicating that the two proteins were glycosylated. Accordingly, deglycosylation with PNGase F resulted in a single sharper band and the molecular weights of E2 and E^rns^ were approximately 40 kDa and 20 kDa, respectively. Thus, the diffuse or multiple bands may represent different glycosylation levels of the proteins. Taken together, these results suggest the two proteins are glycosylated properly.

### Humoral immune responses in rabbits

**Fig. 2:**
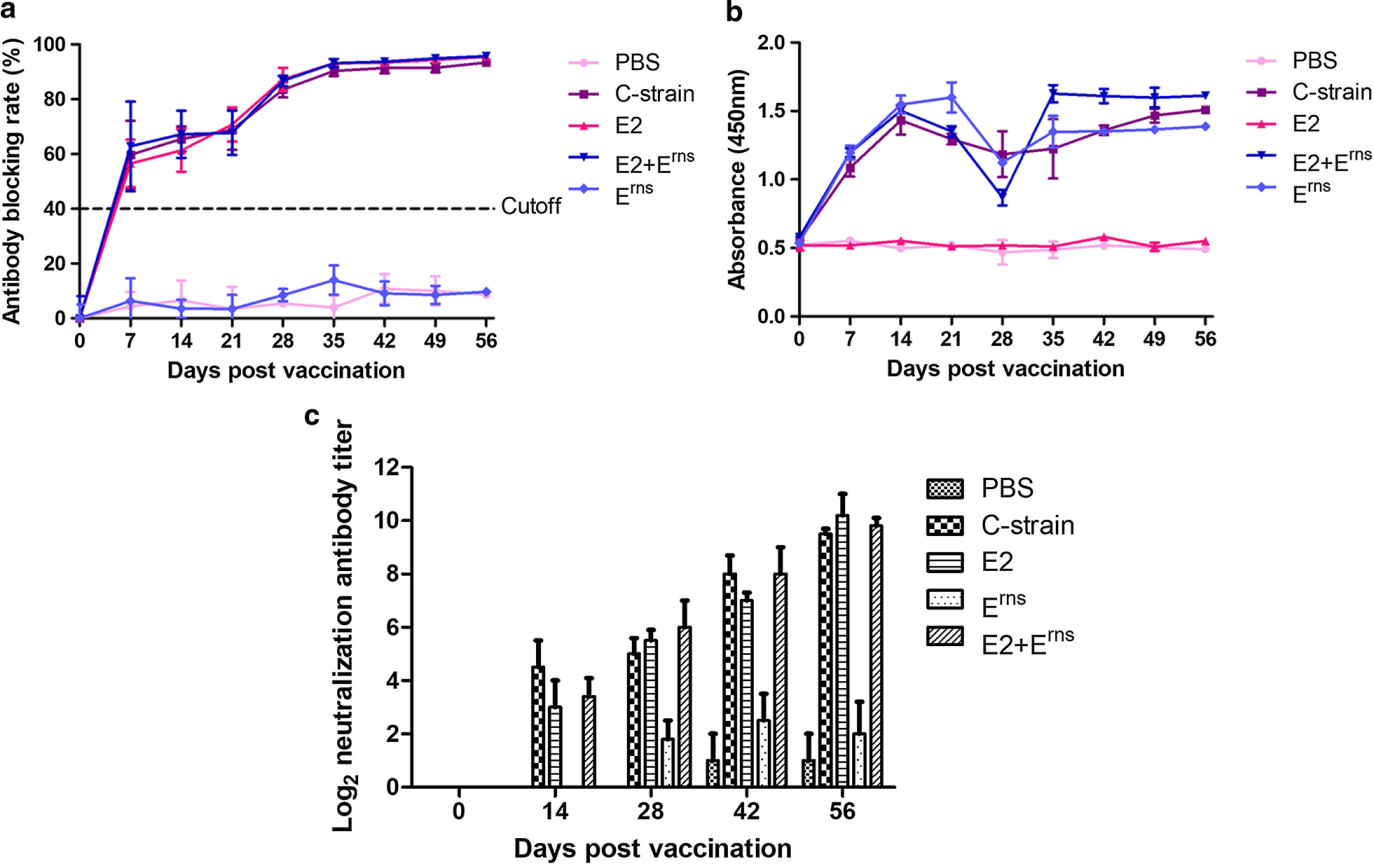
Humoral immune responses in immunized rabbits. Twenty 14-week-old New Zealand white rabbits (four per group) were intramuscularly immunized at days 0 and 28. Serum samples were collected at each week after the first immunization. **a** The presence of E2-specific antibodies was tested using the IDEXX CSFV antibody test kit. The results were reported as the antibody blocking rate and a rate ≥ 40% was interpreted as positive. **b** E^rns^-specific antibodies were detected by ELISA as we described in Materials and Methods. Relative antibody concentrations were determined using the ELISA plate reader at 450 nm. **c** The titers of CSFV specific neutralization antibodies were determined via immunoperoxidase monolayer assay (IPMA) and the results were expressed as the reciprocal of the highest dilution that caused 50% neutralization

To determine the antibodies elicited against CSFV, we analyzed the serum samples for CSFV E2-specific antibodies (Fig. 2a), E^rns^-specific antibodies (Fig. 2b) and anti-CSFV neutralizing antibodies (Fig. 2c). The results of blocking ELISA were reported as the antibody blocking rate and a rate ≥ 40% was interpreted as positive. As shown in Fig. 2a, the rabbits vaccinated with the commercial CSFV vaccine (C strain), CSFV-E2 and CSFV-E2 + E^rns^ developed specific E2 antibody responses as early as 7 d post-immunization (dpi), with mean antibody blocking rates of 59.68%, 56.52% and 62.75%, respectively (cut-off value of 40%). The antibody titers induced by CSFV-E^rns^ were not detected with blocking ELISA. The antibody levels of all vaccinated rabbits peaked at 56 d, with mean antibody blocking rates of 93.40%, 95.47%, and 95.67%, respectively (Fig. 2a). Moreover, it is shown that the rabbits vaccinated with C strain, CSFV-E^rns^ and CSFV-E2 + E^rns^ developed specific E^rns^ antibody responses as early as 7 dpi (Fig. 2b). However, E^rns^-specific antibody was not detected in the CSFV-E2 and PBS groups.

As shown in Fig. 2c, no neutralization activity against CSFV was detected in the PBS group throughout the experiment. All the rabbits vaccinated with the C strain, CSFV-E2 and CSFV-E2 + E^rns^ developed anti-CSFV neutralizing antibody at 14 dpi and peaked at 56 dpi. Besides, the virus neutralization assay (VNA) titers in the three groups are comparable. In contrast, neutralization antibody was not detected in the CSFV-E^rns^ vaccinated rabbits, showing no significant differences with that of the PBS group. Thus, there seems to be close correlation between VNA titers and levels of E2-specific antibodies.

### Cellular immune responses in rabbits

**Fig. 3:**
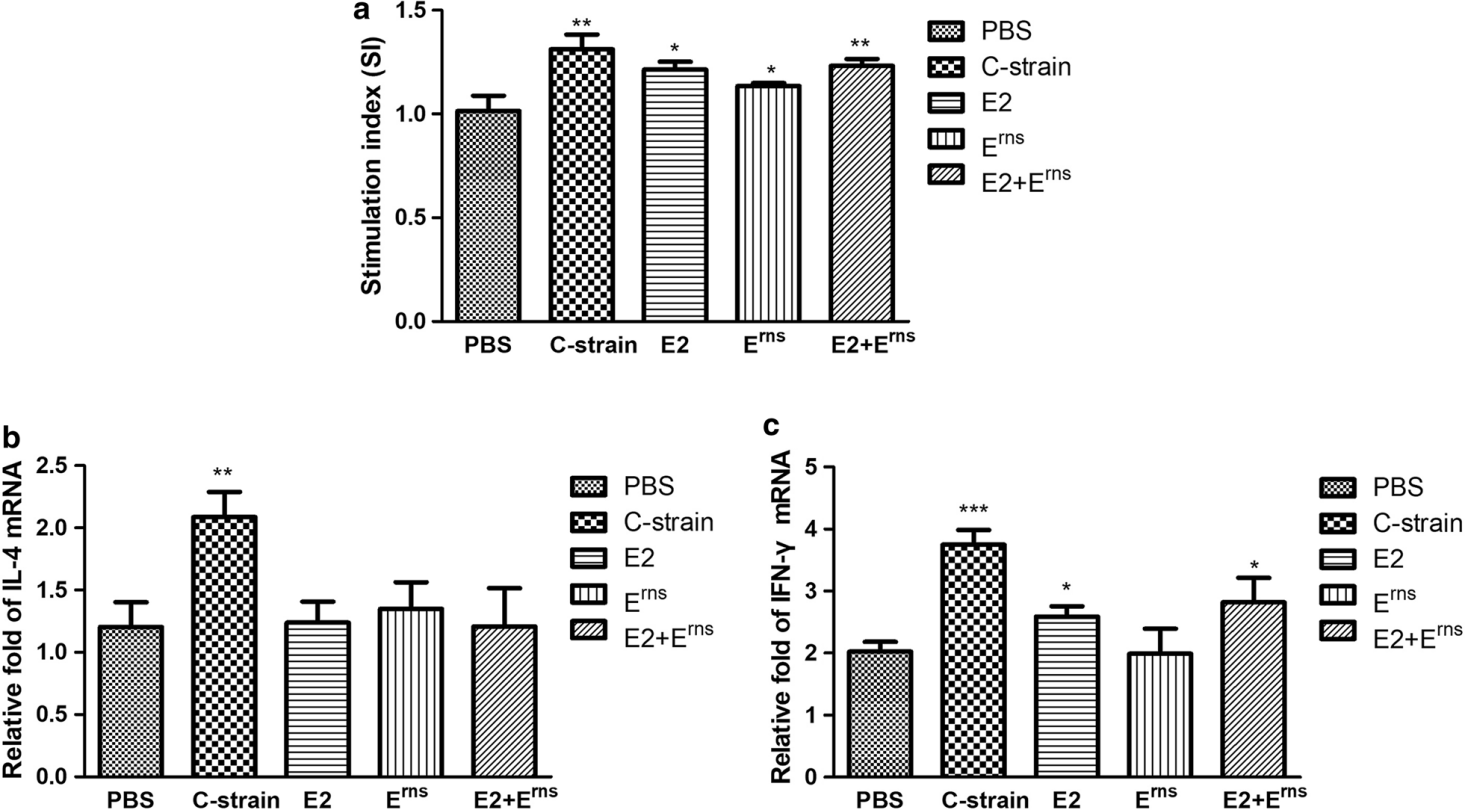
Cellular immune responses in immunized rabbits. PBMCs isolated from the immunized rabbits at 49 days after the primary vaccination were stimulated with or without CSFV Shimen strain in triplicate. The T-lymphocyte proliferation was performed as described in Materials and Methods. The stimulation index **a** was obtained from dividing mean of the OD_450_ values of the CSFV-stimulated wells by mean of the OD_450_ values of CSFV-unstimulated wells. RNA of PBMCs was extracted and subjected to real-time RT-PCR after 72 h of incubation at 37 °C. Relative quantity of IL-4 mRNA expression (**b**) and IFN-γ mRNA expression (**c**) were determined using the expression of the GAPDH gene as the housekeeping gene. Data are shown as mean ± SEM, statistical differences between vaccination groups and PBS groups were measured by one-way ANOVA, indicated by asterisk. *p < 0.05; **p < 0.01; ***p < 0.001

To evaluate the impact of the recombinant protein on cellular immune response, the CSFV-specific lymphocyte proliferative responses were measured at 49 dpi. As shown in Fig. 3a, the stimulation index (SI) of PBMCs from vaccination groups was significantly greater than the PBS group, although the difference is marginal.

To assess the cellular immune responses further, the mRNA expression levels of IFN-γ and IL-4 were evaluated at 49 dpi through real-time RT-PCR. Cytokine IFN-γ is associated with T helper cell type 1 (Th1) responses and cell-mediated immunity. The amount of IL-4 released by the stimulated PBMCs reflected T helper cell type 2 (Th2) responses of the vaccinated groups. All vaccinated groups, except for the PBS group, elicited higher IFN-γ mRNA expression than IL-4 mRNA expression (Fig. 3b, c). These results indicated that the C strain, CSFV-E2, CSFV-E^rns^, and CSFV-E2 + E^rns^ vaccines induced Th1-dominant cellular immune responses in vivo.

### Protection of rabbits against CSFV challenge

**Fig. 4:**
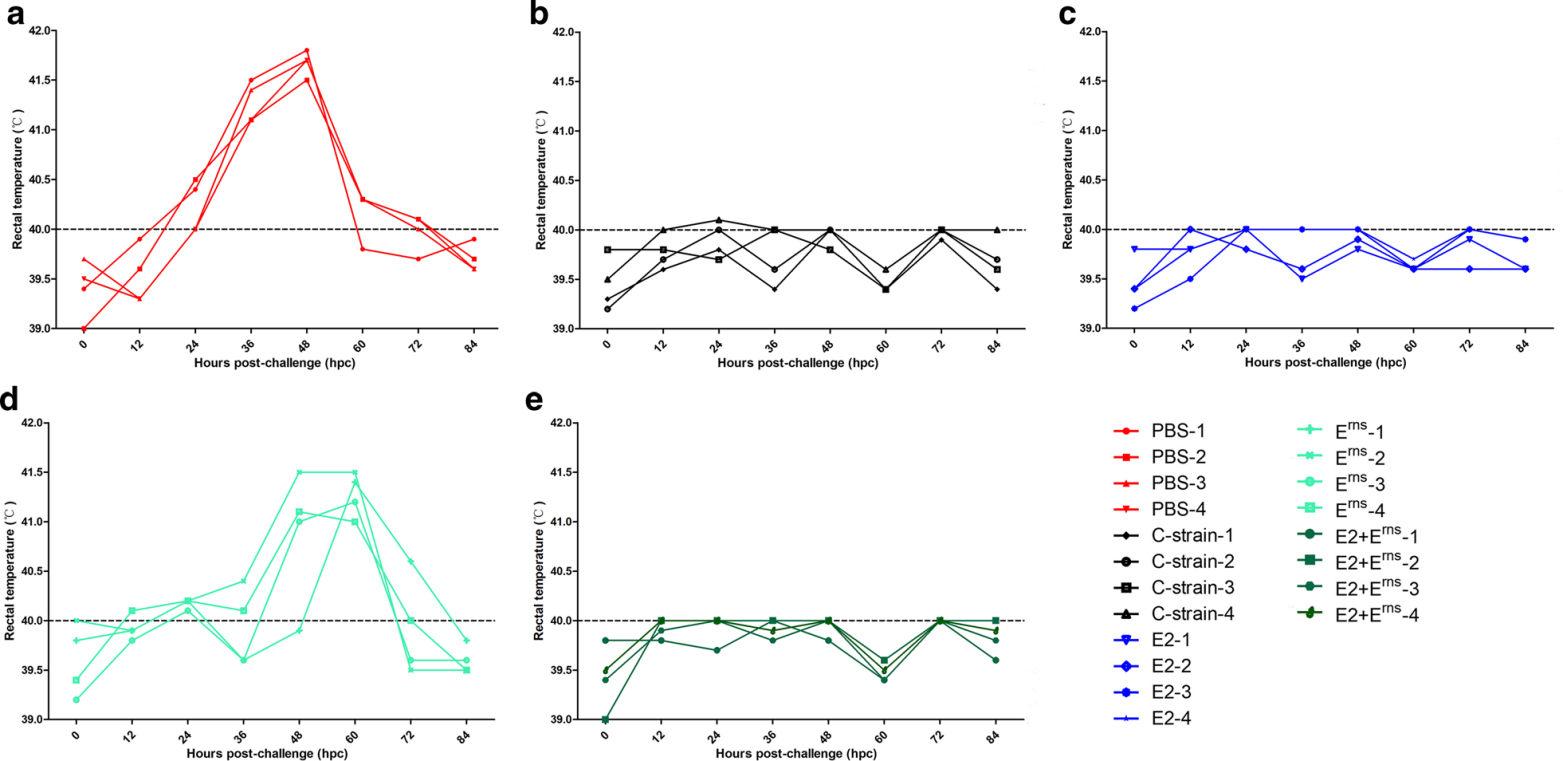
Rectal temperatures of the rabbits after challenge with CSFV C-strain. Rectal temperature was recorded each 12 h-interval throughout the experiment. Fever was considered as rectal temperature ≥ 40 °C and lasting for at least 18 h

All rabbits were intravenously challenged with 100 RID_50_ of CSFV at 4 weeks after booster immunization. The body temperature responses of individual rabbits were measured (Fig. 4). All rabbits in the PBS group displayed typical fever at 24–72 h post-challenge (Fig. 4a). Besides, all rabbits immunized with CSFV-E^rns^ also exhibited typical fever (Fig. 4d). In contrast, the rabbits immunized with C-strain, CSFV-E2, and CSFV-E2 + E^rns^ were completely protected and demonstrated normal body temperature at post-challenge (Fig. 4b, c, e).

**Fig. 5:**
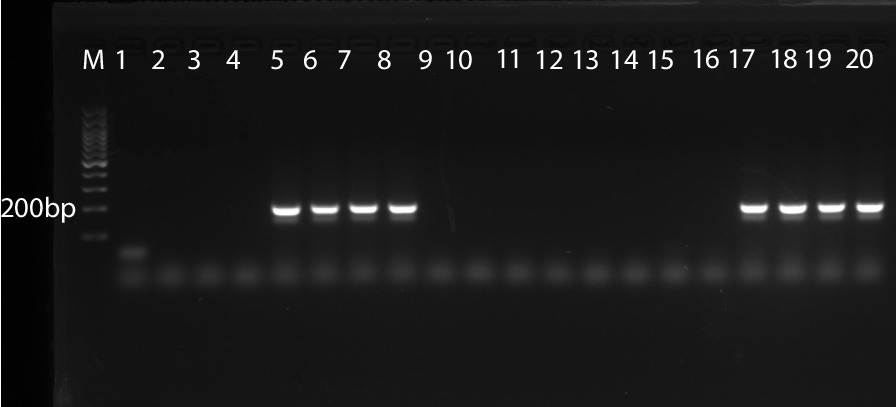
Agarose gel electrophoresis of samples detected by RT-nestPCR assay. The spleens of the rabbits were collected and total RNA was extracted. The viral RNA was detected by the RT-nestPCR amplification. Lane M: DL 100 plus DNA Marker. Lane 1–4: CSFV-E2 group; Lane 5–8: CSFV-E^rns^ group; Lane 9–12: CSFV E2 + E^rns^ group; Lane 13–16: C-strain group; Lane 17–20: PBS group

As shown in Fig. 5, no viral RNA was detected in the rabbits immunized with C-strain, CSFV-E2, and CSFV-E2 + E^rns^. However, viral RNA was detected both in CSFV-E^rns^ and PBS groups. The results are consistent with the observed typical fever in different groups.

## Discussion

In the present study we successfully constructed recombinant baculoviruses expressing E2 and E^rns^. The glycosylation of the two protein is confirmed by PNGase F treatment. We investigated the efficacy of the recombinant E2 and E^rns^ protein as subunit vaccines against CSFV. The results showed that immunization with CSFV-E2 and CSFV-E2 + E^rns^ induced robust humoral immune responses as well as a certain degree of cellular immune responses, which conferred complete protection against the 100RID_50_ CSFV C-strain challenge in the rabbit model. In contrast, immunization with CSFV-E^rns^ could not induce a protective immune response.

In protein engineering, secretory expression is preferred because of cytotoxicity of the target proteins or for easier purification of the expression products with less interference from cellular proteins. Here, high yield of E2 and E^rns^ were obtained from the recombinant baculovirus using the modified pFastBac1 vector containing the gp67 signal peptide. The recombinant protein was secreted into the culture supernatants and could be easily purified using Ni affinity column.

For secretory expression of E2, the transmembrane region was deleted. It was shown that such deletion would not influence the immunogenicity of E2 [19–21]. Although different expression systems have been approached for expression of E2, insect cells maintain many advantages. For instance, insect cells do not produce lipopolysaccharides, which is a concomitant contaminant in proteins expressed in *E. coli*. In addition, insect cells could provide correct protein folding [29] and post-translational modifications, which is particularly important for proteins that are to be used as subunit vaccine candidates. Indeed, it has been confirmed that glycosylation of CSFV envelope proteins is crucial for the immunogenicity [7]. As revealed in the SDS-PAGE and western blot analysis, the E2 protein obtained here is glycosylated efficiently, which would facilitate its immunogenic efficacy.

To avoid aggregation of E^rns^ during secretion and purification, the amphipathic helix at the C terminus that is inserted slightly tilted into the membrane was truncated [25–27]. Moreover, the antigenic structure and organization of E^rns^ protein was analyzed [22–24]. Consequently, the E^rns^ containing the mainly antigenic region was expressed and purified. SDS-PAGE and western blot analysis showed that the E^rns^ protein was efficiently glycosylated in sf21 cells. Furthermore, E^rns^-specific antibody was detected in the C strain, CSFV-E^rns^ and CSFV-E2 + E^rns^ groups, suggesting the obtained E^rns^ protein is immunogenic. The E^rns^ has been shown to be able to induce virus-neutralizing antibodies in the host in previous studies [10–12]. However, in a more recent work, a yeast-expressed E^rns^ failed to induce neutralizing antibodies in vaccinated pigs [18]. In this study, no neutralizing antibody was detected in the E^rns^ vaccinated rabbits either, which is consistent with the recent work. Thus, the glycosylated E^rns^ may not induce high neutralizing antibody titers in vaccinated groups. Since it was reported that a single dose of purified E^rns^ could induce protection against CSFV [7], the exact mechanism mediating the protection remains uncertain.

In this study, we analyzed the CSFV-specific T-lymphocyte proliferative response and cytokine production assay to determine if the proteins induced T-cell responses. All groups produced higher stimulation indices compared with the PBS group. IFN-γ (Th1-type cytokine) and IL-4 (Th2-type cytokine) were measured after stimulation to evaluate the Th1 and Th2 responses. In all the vaccine groups, stimulated T lymphocytes produced a much higher concentration of IFN-γ compared to IL-4. The results indicated that the CSFV-E2, CSFV-E^rns^, and CSFV- E2 + E^rns^ groups may mainly stimulate Th1 response in rabbits.

All rabbits in the PBS group showed typical fever after CSFV challenge. The CSFV-E^rns^ vaccine did not produce protection, as indicated in this group by the typical fever and the presence of viral RNA in the spleen. In contrast, the rabbits of the E2 and E2 + E^rns^ groups were completely protected from challenge infection. These results suggest that E2 protein is essential and may be sufficient for vaccine-mediated protection against CSFV.

## Conclusions

In this study, CSFV E2 and E^rns^ protein were successfully generated by insect cell/baculovirus expression system and the immunogenicity of the proteins was evaluated. There was some controversy about the efficacy of the recombinant E^rns^ protein as subunit vaccines against CSFV. The results presented here supported the conclusion that the E^rns^ could not confer protection against CSFV. In contrast, the CSFV-E2 + E^rns^ and CSFV-E2 are candidate subunit vaccines against CSFV. Thus, our study proved that the glycoprotein E2 is the most immunogenic envelope protein, and alone can protect the rabbits against CSFV in different ways.

## Data Availability

All relevant information is provided in this current manuscript.

## Abbreviations

CSFV: Classical swine fever virus
*E. coli*: *Escherichia coli*
ELISA: Enzyme-linked immunosorbent assay
ANOVA: Analysis of variance
PBMCs: Peripheral blood mononuclear cells
